# Requirements for Becoming an Adjunct Professor in Medicine: A Comparative Analysis of the Regulations of German Medical Faculties

**DOI:** 10.3390/ijerph182211856

**Published:** 2021-11-12

**Authors:** Sarah Altenberger, Roman Leischik, Richard Vollenberg, Ulrich Jehn, Holger Reinecke, Jan Peter Ehlers, Markus Strauss

**Affiliations:** 1 Department of Didactics and Educational Research in Health Science, Faculty of Health, University Witten/Herdecke, 58455 Witten, Germany; sarah.altenberger@uni-wh.de (S.A.); jan.ehlers@uni-wh.de (J.P.E.); 2 Department of Cardiology, Faculty of Health, School of Medicine, University Witten/Herdecke, 58455 Witten, Germany; roman.leischik@uni-wh.de; 3 Department of Medicine B, Gastroenterology and Hepatology, University Hospital Muenster, 48149 Muenster, Germany; richard.vollenberg@ukmuenster.de; 4 Department of Medicine D, Division of General Internal Medicine, Nephrology and Rheumatology, University Hospital of Muenster, 48149 Muenster, Germany; ulrich.jehn@ukmuenster.de; 5 Department of Cardiology I—Coronary and Peripheral Vascular Disease, Heart Failure Medicine, University Hospital Muenster, Cardiol, 48149 Muenster, Germany; holger.reinecke@ukmuenster.de

**Keywords:** professorship, academic career, career in medicine, research in medicine, education of healthcare professionals

## Abstract

Background: Following a medical habilitation or equivalent qualification after continuous scientific activity, one can apply for a position as an adjunct professor (außerplanmäßige Professur). The medical faculties in Germany have issued regulations for these appointments. The aim of this paper was to compare the requirements for appointment as an adjunct professor among medical faculties. Methods: The currently valid regulations of medical faculties in Germany were analyzed for the target criteria of publication performance, teaching performance, possibility of shortening the procedure; consideration of appointment for junior professor, patents, acquisition of third-party funding, medical didactic qualifications, and/or special scientific achievements; and review procedure. Results: An analysis of 38 currently valid regulations showed large differences between the requirements. The number of required first/senior authorships differs significantly within the regulations (from 4 to 16). The median of the required number of first/senior authorships is six (Q1 = 5, Q3 = 7). In total, 93% (*n* = 35) of the universities provide information on the publication medium or the value of the publication. Third-party funding is desired or required in 68% (*n* = 26) of the regulations. There are also clear differences in the scope of required teaching activities, which range from two to a maximum of six years of teaching. Shortening the time to apply for an adjunct professorship is possible in 45% (*n* = 17) of the cases. In total, 97% (*n* = 37) of the faculties provide information on external review, with 71% (*n* = 27) most frequently requesting one or two external reviews. Conclusion: The regulations show clear differences among individual requirements for adjunct professorship. Standardization would be desirable and would lead to comparable conditions and therefore also to a fair recognition of scientific achievements.

## 1. Introduction

In addition to appointment as a university professor or chair, adjunct professor (außerplanmäßige Professur, or apl. Professur) is one of the highest levels of a university career in Germany [1]. The requirements are defined by the regulations of the medical faculties and are issued in accordance with the Higher Education Acta (Hochschulgesetze) of the individual federal states. In contrast to a full professorship (Ordentliche Professur), the title is not linked to the filling of a position and does not automatically establish an employment relationship. In addition, it is not possible to distinguish the title of adjunct professor from full professor, because the prefix “apl.” is not obligatory.

An international classification of adjunct professorship as an academic career path is only possible to a limited extent. Previous studies show clear differences in the regulations for professorships in the stipulated requirements among nations and faculties [2,3,4]. In view of the various academic career paths worldwide, there are calls for more transparency and better comprehensibility of the career stages in the international scientific community [5]. The aim of this manuscript was, therefore, to provide a first comparative overview of the currently valid requirements for becoming an adjunct professor (apl. Professur) in Germany.

## 2. Methods

### 2.1. German System

In Germany, there are several ways to become an adjunct professor. One possibility for obtaining an adjunct professorship is habilitation, which is considered a quality criterion for achievements in teaching and research and proof of “Lehrbefähigung” (venia legendi) and confers the academic degree of “Privatdozent” (PD, Priv.-Doz.) [6]. In addition to habilitation, becoming a junior professor (Juniorprofessur) is another option. The junior professorship was established by the fifth amendment to the German Higher Education Framework Act (Hochschulrahmengesetz) in 2002 with the intention to make scientific careers more attractive and competitive in international comparison [5]. The reasons for this were, on the one hand, to prevent the emigration of German scientists and, on the other hand, to ensure more security and predictability for young academics [7,8].

### 2.2. Study Design

The valid regulations of the medical faculties in Germany as of 16 July 2021 were included. For this purpose, the relevant regulations were downloaded from the websites of the respective medical faculties. In case of missing information, the dean’s office of the respective university was contacted in writing.

### 2.3. Inclusion Criteria

The inclusion criterion was the existence of the regulation for adjunct professorship at an accredited German medical faculty as of 16 July 2021.

### 2.4. Exclusion Criteria

The exclusion criterion was the absence of a currently valid regulation for adjunct professorship at an accredited German medical faculty.

Cooperative projects between German hospitals and foreign medical faculties with the attainment of a foreign degree were not included in the analysis.

### 2.5. Target Criteria

**Figure 1:**
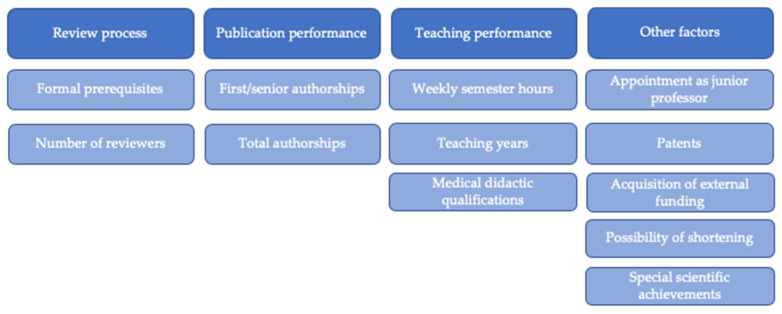
Target criteria for the attainment of an adjunct professorship in Germany.

The regulations were analyzed for the following target criteria (Figure 1): Publication performance: Number of first/senior authorships, co-authorships, total authorships, and consideration of value; teaching performance: Extent of teaching performance (teaching years/weekly semester hours (SWS)) and possibility of shortening the procedure; consideration of: Appointment as junior professor, patents, acquisition of external funding, medical didactic qualifications, and/or special scientific achievements; review procedure: Formal prerequisites for reviewers and number of reviewers.

### 2.6. Goal of Analysis

The aim of this paper was to analyze and compare interfaculty requirements for an adjunct professorship based on the above target criteria, taking into account the regulations of each university.

### 2.7. Statistical Analysis

The target criteria publication performance (total authorship, first/senior authorship, and co-authorship) and teaching performance (number of years) are described by the maximum (max), minimum (min), median (quartile 2), quartile 1 (Q1), and quartile 3 (Q3).

The criteria number of reviewers, appointment as Juniorprofessur, shortening the procedure, patents, raising third-party funds, medical didactic qualification, and special scientific achievements are given in absolute and relative counts.

## 3. Results

Finally, 38 regulations of the 43 existing faculties in Germany could be included in the analysis (corresponding to 88%). Medical faculties with valid appointment regulations are listed in Supplementary Table S1. Those medical faculties without regulations for adjunct professorship were as follows: University Augsburg, University Bielefeld, Medical School Berlin, Medical School Potsdam, and Medical School Hamburg.

### 3.1. Basic Requirements

The prerequisites for becoming an adjunct professor are continuous outstanding scientific and teaching activity and completion of a prior qualification process. This is proven if the applicant has held a junior professorship, has undergone habilitation, or has achieved equivalent scientific achievements [9,10,11,12,13,14,15]. A successful doctorate is usually a necessary criterion for a junior professorship or habilitation (this is often listed in the habilitation regulations of the medical faculty). All regulations have in common that applicants must have proven themselves in research and teaching. After having successfully completed the qualification phase, one can submit an application for an adjunct professorship to the associated faculty.

**Figure 2:**
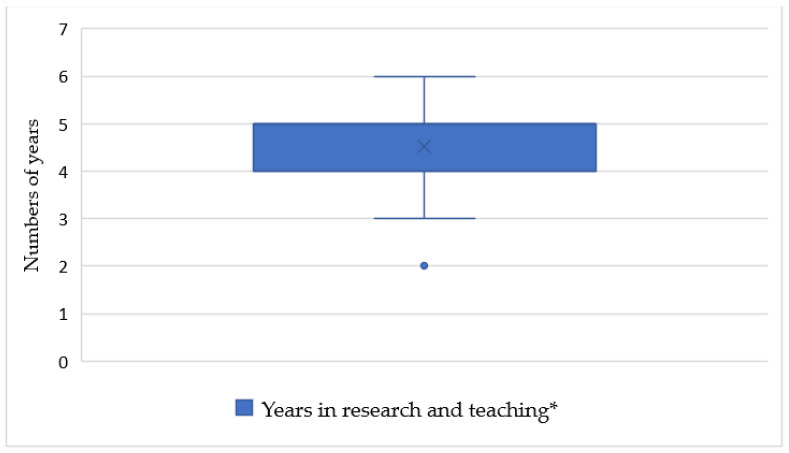
Distribution of number of teaching years required to obtain adjunct professorship at German medical faculties. * Required years in research and teaching in the individual regulations (faculties considered, *n* = 38).

**Figure 3:**
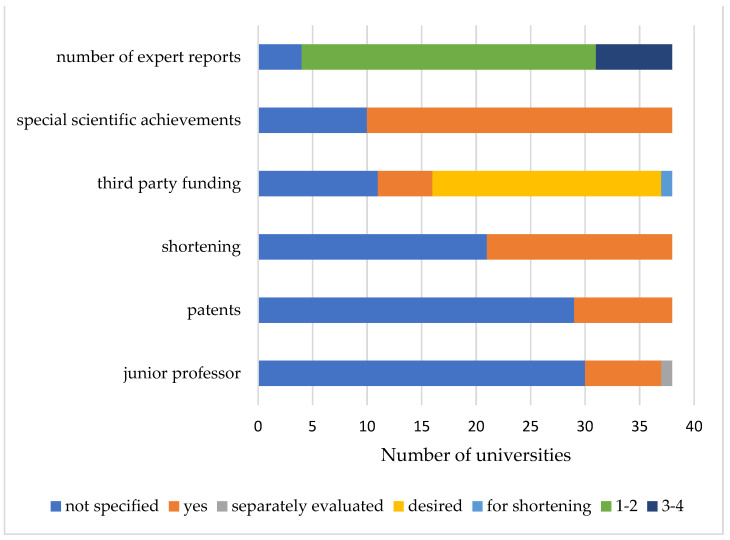
Relevant factors for obtaining an adjunct professorship at German medical faculties.

The period after habilitation is considered, although the required period of continued teaching and research years until the possibility of application varies (Figure 2). Among the regulations, 53% of the universities (*n* = 20) specify a period of five to six years (Q1 = 4; median = 5; Q3 = 5), 29% (*n* = 11) require three to four years, and 11% (*n* = 4; min = 2) require only two years. Three universities (8%) do not provide any information. In cases where applicants can demonstrate special scientific achievements, a shortening of the time for application is mentioned in the regulations at many universities (*n* = 17, 45%). This is illustrated schematically in Figure 3 In six regulations (18%), attaining a listed position can lead to a shortening of the time to nomination as an adjunct professor. For example, at Ruhr University of Bochum, a reduction from five to four years is possible if the applicant has achieved a place on the list for a W2 or W3 professorship or has received higher scientific honors (e.g., Leibniz Prize) [16]. In 21 regulations (55%), no information on the possibility of shortening the time is provided (Figure 3).

### 3.2. Research Performance

**Figure 4:**
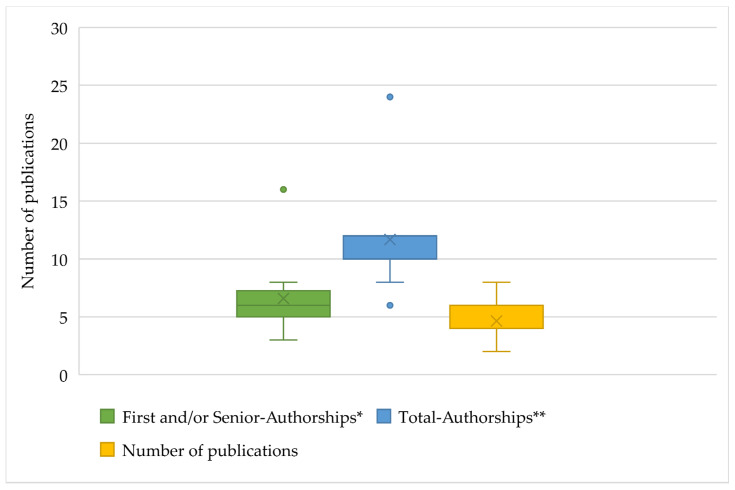
Distribution of publication performance required to obtain adjunct professorship at the German medical faculties. * Required first and/or senior authorships in the individual regulations (faculties considered, *n* = 34). ** Required total authorships in the individual regulations (faculties considered, *n* = 34).

The assessment of research performance is usually based on the number and value of published papers. In the assessment, the regulations distinguish between the numbers of first and senior authorships, co-authorships, and total publications. The analysis shows differences in the required minimum number of publications (Figure 4). For 53% of the regulations (*n* = 20), five to seven publications with first and senior authorships are most frequently required (Q1 = 5; median = 6; Q3 = 7.25). In addition, statements such as “predominantly” first/senior authorships or “continuous publication achievements” are found. The lowest number of required first/senior authorships is four (*n* = 4.11%) and the highest is eight (*n* = 6.16%). This usually refers to the time after habilitation has been completed. At only at two universities (5%), the time from the doctorate is evaluated, and in this case, 16 first/senior authorships are required. The University of Leipzig also distinguishes between clinical subjects (eight first/senior authorships) and non-clinical subjects (12 first/senior authorships) [17]. Senior authorships are considered equivalent to first authorships in 79% (*n* = 30) of the cases and are evaluated as such.

Because 15 universities (39%) do not provide any information about the number of co-authorships, it is also only possible to make a statement about the desired number of total publications for 19 universities (49%). Of the universities that provide information on co-authorship, 43% (*n* = 10) require a total output of ≤10 publications. Two universities require >20 total publications, but it should be noted here that these are the universities mentioned above that require 16 first/senior authorships from the “promotion“ onward.

In general, only publications that have undergone a peer-review process and have been published in a peer-reviewed journal are considered. Evaluation of the value of publications in scientific journals is based on the Science Citation Index of the Journal Citation Report (SCI/JCR) of the Institute for Scientific Information (ISI). The evaluation of publication performance is mentioned in 31 regulations (82%).

Some regulations require publications in a journal to belong to a certain ranked percentage. This ranking is formed based on the impact factor of journals in each subject group. Table 1 shows examples of the criteria used to assess the value.

Four faculty regulations (11%) use a point scoring systems that assigns scores to publication performance based on the impact factor. However, the scoring systems are not uniform, which makes it difficult to compare them. The achieved scores are summed up and a certain sum is declared as a “target limit”.

### 3.3. Performance in Teaching

The regulations present a more uniform picture regarding the required semester hours per week (SWS). On average, most universities require between one and two SWS (*n* = 27, 72%). Only four faculty regulations (11%) do not provide any information in this regard. On average, this amount is required for five to six years (*n* = 20, 53%). Eleven universities (29%) require three to four years, and four (11%) require only two years. In 8% of the faculty regulations (*n* = 3), no detailed information is given. Four regulations (11%) assess teaching by an individual point system and three (8%) state the number of teaching hours “to be regulated separately”. As an example, the University of Brandenburg requires 60 teaching hours within the last four years [9].

Student evaluations of the quality of courses are also considered in some regulations. For example, 14 faculties (37%) require a teaching evaluation. The Johannes Gutenberg University Mainz also requires a grade point average of at least 2.5 in the teaching evaluations of the last three years [11]. Supervision of Bachelor’s theses, Master’s theses, or doctoral dissertations is also desired (*n* = 1, 3%) or required (*n* = 20, 53%). At present, Carl-von-Ossietzky University of Oldenburg requires at least three supervised doctoral theses that have either been completed or are currently in the supervision process [18].

In this context, it is worth mentioning that three universities explicitly require participation in didactic training in their regulations [12,15,19]. At Heinrich Heine University Düsseldorf, an additional full-day advanced training course on ensuring good scientific practice (Gute Wissenschaftliche Praxis) is required [19].

At the University of Regensburg, the total number of required publications can be reduced by two if special achievements in teaching can be proven [20].

### 3.4. Assessment

After the opening of the procedure, the submitted documents are first subjected to a formal examination for correctness and completeness. This is usually achieved by a commission set up by the university for this purpose. At this point, 37 (97%) of the universities initiate an external review by obtaining expert opinions. The majority (*n* = 27, 73%) require one or two external reviews and 19% (*n* = 7) require three to four.

### 3.5. Further Criteria

Additional factors include scientific achievements such as book contributions, organization of congresses, preparation of casuistry, and awards with scientific prizes. Nomination for list placement is specified in 18 faculty regulations (53%). In 26 regulations (68%), the list placement for a W2 or W3 professorship is explicitly stated; in these cases, this often results in the possibility of a shortened procedure for an adjunct professorship. A further criterion is the acquisition of third-party funding or the successful registration for patent protection (Figure 3).

At the Technical University of Munich, exemplary third-party funds can lead to a shortening of the adjunct professorship procedure [21]. It is worth mentioning that the successful acquisition of third-party funding is desired or taken into account in many regulations (*n* = 18, 48%). At five universities (13%), it is even a required. At 26 universities (76%), the consideration of patents is not included in the evaluation. At 30 universities (79%), the regulations do not indicate whether a junior professorship is considered in the adjunct professorship procedure. At the University of Ulm, the procedure for awarding an adjunct professorship can be carried out during the final evaluation of a junior professor [14]. Georg-August-University of Göttingen indicates that it separately regulates the procedure for awarding the title adjunct professor to junior professors who have successfully completed evaluation after the end of the junior professorship [22].

## 4. Discussion

In the past, the research conditions in Germany led to an emigration of scientists to other countries (especially to the USA) [5]. Criticism was already leveled at the time at poor plannability and the lack of transparency in the conditions. Measures were taken to make Germany more attractive again as a location for science. These included the introduction of the junior professorship. Our study focused on making the conditions of an academic career in Germany more transparent for German and foreign applicants.

In German-speaking countries, the adjunct professor position is a recognition of special achievements in science and teaching. The title is based on prior qualifications, usually habilitation or equivalent achievement. Habilitation is regarded as a quality criterion for research and teaching achievements and as proof of “Lehrbefähigung” (venia legendi) in the respective subject area [6]. In addition to habilitation, there are now equivalent scientific and didactic qualifications that are accepted as prerequisites for an adjunct professorship. At the Goethe University Frankfurt am Main, for example, applicants who have held a junior professorship can apply for an adjunct professorship [10]. Since the title of adjunct professorship is unique to Germany, it is only awarded at German universities. Therefore, equivalent international comparability and classification of it are difficult.

In the USA, the academic career path can lead to a “full” professorship by following three stages and the so-called tenure track. After a period of usually seven years as an assistant professor, promotion to associate professor is possible within the framework of the tenure track. The associate professorship is linked to specific university requirements such as the acquisition of third-party funding, outstanding achievements in research, and teaching and clinical activity, and corresponds roughly to the adjunct professorship in Germany [2,23].

The junior professorship in Germany is limited in time. This means that it is difficult to plan for the future, because there is no guarantee of getting a full professorship afterward. In order to ensure a predictable path to permanent professorship, the German Scientific Council (Deutscher Wissenschaftsrat) in 2014 advocated for further development of the junior professorship into a tenure track position [24]. Therefore, to make academic careers in Germany more attractive again, the tenure track program was introduced in 2017, based on the American tenure track, and was initially designed for 15 years. If the evaluation of the probationary period is positive, the professorship will be made permanent [25]. This means that after achieving a junior professorship, the time-consuming appointment procedure for a full professorship is no longer necessary [25].

In Great Britain, one climbs the career ladder starting as a lecturer, which is comparable to the assistant professor in the USA and the “Privatdozent” in Germany. This temporary position is characterized by activity in research and teaching. After three to four years, this position can be made permanent, and a promotion can be attempted. The next career level is senior lecturer or reader. Senior lecturer is comparable to associate professor in the USA, while the reader can be considered a full professor without a chair in Germany (W2 Professur). Within the last 10 years, the title of “reader” or “senior lecturer” has been replaced by the title “associate professor” at some British universities. This is particularly intended to improve international recruitment. However, the appointment is initially only valid for five years and will only be made permanent after a positive evaluation. The highest academic degree in the U.K. is full professor, which is comparable to a W3 Professur (chair) in Germany. Thus, there is a heterogeneous picture and a lack of international comparability. This fact is also still evident in Germany.

Within the last 20 years, studies have repeatedly shown large differences in the requirements for academic qualification [2,3,4]. This is still evident, although changes are discernible in some places. Nevertheless, in addition to the differences in individual evaluation criteria, imprecise formulations lead to more scope for individual assessment and consideration by the commissions and experts. This scope of judgment works against uniformity on the one hand and transparency in the award procedures on the other.

This suggests that the adjunct professorship regulations are understood more as a guideline at some faculties, similar to what was assumed in the work of Nagelschmidt et al. [26] for habilitation regulations.

New, or at least not mentioned in previous papers, are point systems at four medical faculties. These scoring systems are not uniform, so they can be compared to one another only to a limited extent. The introduction of a nationwide scoring system would be a feasible solution for the standardization of adjunct professorship regulations.

It should be noted that an increasing appreciation of didactics and teaching has found its way into the regulations. In addition to course evaluations, many universities now require proof of didactic training. In the year 2000, there was no information about such criteria in the evaluation of applicants [3]. The requirement for didactic qualification was first described by Sorg et al. [4] in a review paper in 2012. At this time, six universities already required course evaluations [4].

Due to the fact that many habilitation regulations now require didactic qualifications, applicants already bring the relevant didactic basics with them on this path [27]. The supervision of theses has also received increasing attention over the past 20 years. Specifically, this was used as a criterion in 2012 and at 21 faculties to date. In contrast, only 12 faculties required this in 2000 [3,4].

Information regarding the required teaching performance of the applicants was mentioned more precisely in the regulations. While in 2000, only 18 of the 37 faculties with valid regulations provided information on the extent of teaching performance, in 2012, the number had risen to 28 (80%) [3,4]. At the current time, only four faculties (11%) do not specify the scope of teaching in their regulations. Most universities also specify the scope of teaching in the form of semester hours per week (72%, *n* = 27). In contrast, in 2000, only eight universities provided information on the number of SWS [3]. This development regarding concrete information on teaching performance is welcome and ensures better interfaculty comparability.

Concerning the review process, the majority of faculties continue to use external reviewers. The number of faculties requesting external evaluations has increased continuously since 2000, from 27 to the current 34 [3]. This development is contrary to the demands of Pabst et al. [3], who suggested doing without external expert opinions in order to relieve colleagues in the field of the responsibility of preparing such opinions.

A continuous increase over the last 20 years can also be seen in the demand for acquired third-party funding. From five faculties that took this into account in 2000, the number has risen to a total of 23 (61%) universities that demand or desire the acquisition of third-party funding [3].

The requirements for publication performance are still not very uniform among the various regulations. However, a process of harmonization has become apparent in recent years. At the present time, only 6% (*n* = 2) still make imprecise statements, such as “predominantly first/senior authorships” or “continuous publication achievements”. In contrast, in 2000, the number of such imprecise statements was significantly higher and were mostly limited to statements such as “publications in peer-reviewed and internationally recognized journals” [3]. However, in 2012, 51% of the regulations provided concrete information on publication performance [4]. It can be seen that a large part of the requirements have been specified.

Although the number of desired first/senior authorships has stayed constant since 2012 and is still six on average, there are still large interuniversity differences. These are most apparent in detailed requirements such as number and significance of journals considered [4]. If we look at total authorships (first/senior and co-authorships) as an example, the range extends from a minimum of six to a maximum of 24 required publications (however, the period taken into account varies among regulations). An assessment of value based on different evaluation instruments is carried out by 93% of faculties. Mostly, the impact factor is used for this purpose. However, it should not go unmentioned that the impact factor also receives a critical appraisal [28,29]. The basis of the impact factor is the citation frequency, which is primarily reflected in the size of the corresponding research area. This results in a disadvantage for specialized journals in rather small medical fields. Therefore, the main criticism is to use the impact factor for an interdisciplinary comparison [30].

Some universities try to take this point of criticism into account by setting the sums of the impact factors in relation to the value of a journal in the corresponding area of expertise. Several regulations do not address this point, so an adjustment and standardization would be urgently needed here. The Working Group of Scientific Medical Societies (Arbeitsgemeinschaft der Wissenschaftlichen Medizinischen Fachgesellschaft, AWMF), in a statement, distanced itself from the impact factor as an evaluation standard. Instead, there is a call for multidimensional assessment by means of objectifiable criteria [31]. Nevertheless, this has not been considered in the regulations.

As already mentioned above, the path to an adjunct professorship is no longer just possible only through habilitation. At some faculties, equivalent qualification processes, for example, an appointment to a junior professorship, are alternatives. This category was introduced in 2002 with the fifth amendment to the German Higher Education Framework Act (Hochschulrahmengesetz) and, as an alternative to habilitation, was intended to provide a further qualification path toward a lifetime professorship. Nevertheless, a survey by Sorg et al. [32] of members of habilitation committees in 2016 made clear that habilitation still had a high value, even if the chances of obtaining a chair afterward were rated as only mediocre to low. To date, there are no comparable surveys on the importance and valuation of the adjunct professorship. However, it can be assumed that the appreciation for the position is equivalent to that of habilitation. On the one hand, the significant increase in requirements in the regulations indicates the importance of the adjunct professors. On the other hand, it is a beneficial career factor that is associated with a certain reputation advantage. Alawi et al. [2] placed the adjunct professor below a full professor (W2/W3). This is justified by the fact that it is an unpaid faculty position; in contrast, a full professor is associated with a management function and personnel responsibility. A survey regarding the need to reform the adjunct professorship regulations as they exist with regard to the habilitation regulations does not yet exist [32]. It can be assumed that the desire for uniform regulations nationwide, standardization and more transparency could also apply to regulations for the adjunct professor position.

Moreover, it is regrettable that data have not yet been collected on the number of adjunct professorships and the proportion of women. An estimation can therefore only be made approximately based on other data. The number of habilitations in the field of medicine has remained at a stable level over the past few years [33]. It can be assumed that the next step on the academic career ladder for habilitated professors is to strive for an adjunct or full professorship. Therefore, it seems possible to classify the number of adjunct professors in relation to the number of habilitations. The question of the proportion of women allows further conclusions, because the proportion has risen steadily in recent years. The proportion of female habilitation candidates rose from 18% in 2006 to 32% in 2019 [33,34]. The number of female full professors has increased steadily in recent years, reaching 25% of all full-time professors in medicine in Germany in 2019 [33]. It can therefore be assumed that the proportion of female adjunct professors has also increased.

## 5. Conclusions

The analysis of the regulations for adjunct professorship at medical faculties continues to show a clear heterogeneity in the requirements for applicants. This is particularly evident in the area of required publication performance, which differs significantly among individual regulations in both the number and the consideration of value; however, there is also large variability in the required number of teaching years and the possibility of shortening this time. Furthermore, imprecise formulations in some regulations lead to a certain scope for interpretation and thus do not allow for transparency in the award procedure. For these reasons, general interuniversity and intrauniversity comparability is often not possible. It can also be seen that the demands placed on young academics have risen steadily over the years.

Despite criticisms of the status quo, efforts to improve can be seen within the past 20 years. This is reflected in the consideration of teaching quality and required medical didactic training. Ultimately, there is still room for improvement in order to standardize the requirements among faculties and thereby bring about a desirable increase in transparency in this qualification process.

## Figures and Tables

**Table 1:** Examples of assessment details in the context of publication performance required by individual universities.

Value Details	%	Number
I. and II. Quartile of area of expertise *	3%	1
>5 times the IF of respective area of expertise **	3%	1
IF of at least 10 ***	3%	1
IF > 40 since habilitation ****	3%	1
12 times median *****	6%	2
Detailed consideration ******	32%	10

* Original papers must be published in international or internationally accepted peer-reviewed journals that are ranked in the first or second quartile of their area of expertise (criterion of Charite University Berlin). ** The sum of the impact factors of original papers must be >5 times the impact factor of the discipline average (according to the current Journal Citation Report) (criterion of University Greifswald). *** At least three papers with first/senior authorship in journals with a discipline-specific reputation with an impact factor of at least 10 (criterion of University Freiburg). **** For all original papers after habilitation, the impact factor should be >40 or cumulative citation frequency >120 or three papers as first/senior author in the top group of the discipline (criterion of Ruhr University Bochum). ***** The sum of the impact factor points of publications must be equal to 12 times the median of the corresponding area of expertise in the latest SCI Journal Citation Report (criterion of University Bonn, RWTH Aachen University). ****** Detailed consideration, assessments by classification in categories or grids, assessment by point systems.

## Data Availability

The datasets analyzed during the current study are available from the corresponding author upon reasonable request.

## Supplementary Materials

The following are available online at https://www.mdpi.com/article/10.3390/ijerph182211856/s1, Table S1: Universities in Germany with currently valid adjunct professorship regulations.
