# Supplementary material for: Requirements for Becoming an Adjunct Professor in Medicine: A Comparative Analysis of the Regulations of German Medical Faculties
Source: Int J Environ Res Public Health. 2021 Nov 12;18(22):11856. doi: 10.3390/ijerph182211856 (PMC8621888; doi:10.3390/ijerph182211856)
Supplement: Supplementary file 1 [file ijerph-18-11856-s001.zip › ijerph-1406680-supplementary.pdf]

Table S1. Universities in Germany with currently valid adjunct professorship regulations.

RWTH Aachen University.	Friedrich-Schiller-University Jena
Charite-University Medicine Berlin	Christian-Albrechts-University zu Kiel
Ruhr-University Bochum	University of Cologne
Rheinische-Friedrich-Wilhelms-University Bonn	University Leipzig
Brandenburg Medical School Theodor Fontane	University Lübeck
Technical University Dresden	Otto-von-Guericke-University Magdeburg
Heinrich-Heine-University Düsseldorf	Johannes-Gutenberg-University Mainz
University Duisburg-Essen	Philipps-University Marburg
Friedrich-Alexander-University Erlangen-Nürnberg	Ludwig-Maximilians-University Munich
Goethe University Frankfurt am Main	Technical University of Munich
Alberts-Ludwigs-University Freiburg	Westfälische Wilhelms-University Münster
Justus-Liebig-University Gießen	Carl von Ossietzky University Oldenburg
Georg-August-University Göttingen	University Regensburg
University Greifswald	University Rostock
Martin-Luther-University Halle-Wittenberg	University des Saarlandes
University Hamburg	Eberhard-Karls-University Tübingen
Medical School Hannover	University Ulm
Ruprecht-Karls-University Heidelberg	University Witten/Herdecke
Ruprecht-Karls-University Heidelberg Medical Faculty Mannheim	Julius-Maximilians-University Würzburg
